# Physically active primary care doctors are more likely to offer exercise counselling to patients with cardiovascular diseases: a cross-sectional study

**DOI:** 10.1186/s12875-022-01657-3

**Published:** 2022-03-29

**Authors:** Christine Shamala Selvaraj, Nurdiana Abdullah

**Affiliations:** Department of Primary Care, University of Malaya, Kuala Lumpur, Malaysia

**Keywords:** Physical activity, Primary care doctors, Counselling, Chronic disease, Cardiovascular diseases

## Abstract

**Background:**

Regular physical activity had been shown to reduce morbidity and mortality from chronic diseases such as cardiovascular diseases, hypertension, type 2 diabetes mellitus, dyslipidaemia, obesity/metabolic syndrome, osteoarthritis, osteoporosis, bronchial asthma and chronic obstructive pulmonary disease. Research had shown that physically active doctors were more likely to offer exercise counselling to patients. However, few studies looked into this association with counselling practices to patients with specific chronic diseases. This study aims to determine the association between physical activity levels of primary care doctors (PCDs) in Malaysian private practice with physical activity counselling to patients with chronic diseases.

**Methodology:**

A cross-sectional study involving PCDs in private practice in 3 states was done. Participants were recruited from members of the Malaysian Academy of Family Physicians and attendees of a conference. A self-administered questionnaire obtained information on sociodemography, initiation of exercise counselling to patients with chronic diseases as well as physical activity levels using the International Physical Activity Questionnaire (IPAQ).

**Results:**

The response rate was 32.3% (272/842). 47.1% of the respondents were post-graduate holders. 50% of participants had a moderate level of physical activity and 24.3% a high level. Most respondents answered ‘always’ or ‘often’ for initiation of exercise counselling to patients with cardiovascular diseases (59.9%), hypertension (72.8%), type 2 diabetes mellitus (78.6%), obesity/metabolic syndrome (86.4%), dyslipidaemia (81.6%), osteoarthritis/osteoporosis (41.9%) and bronchial asthma/COPD (29.5%). PCDs being physically active and non-smokers were associated with a higher initiation of exercise counselling to patients with cardiovascular diseases. Doctors with post-graduate degrees were more likely to offer exercise counselling to hypertensive patients.

**Conclusion:**

The association between PCDs’ physical activity levels and their physical activity counselling varies between different types of chronic diseases. Primary care doctors with higher physical activity levels were more likely to initiate physical activity counselling in patients with cardiovascular disease during chronic disease follow up visits.

**Supplementary Information:**

The online version contains supplementary material available at 10.1186/s12875-022-01657-3.

## Introduction

Prevention is one of the building blocks of primary care and nothing paves the way for that like a healthy lifestyle. Apart from a balanced diet and controlled levels of stress, physical activity constitutes an extremely important part of a healthy lifestyle. Regular physical activity is vital for well-being and health for people of all ages as it has been shown to reduce morbidity and mortality from many chronic diseases [1].

Chronic diseases, which are also known as non-communicable diseases (NCDs) caused 71% of all deaths globally in 2016 and 73% of all deaths in Malaysia in 2014 [2–4].

Physical inactivity is one of the behavioural risk factors causally linked with cardiovascular diseases, chronic respiratory disease, cancer and diabetes and it can lead to changes in metabolic profiles which include elevated blood pressure, increased glucose and lipid levels as well as overweight/obesity [5]. Regular physical activity had been shown to benefit those with chronic diseases such as cardiovascular diseases (ischaemic heart disease and stroke), hypertension, diabetes mellitus, obesity, metabolic syndrome, dyslipidaemia, osteoarthritis, osteoporosis, asthma and chronic obstructive pulmonary disease [6].

Primary care doctors (PCDs) have the advantage of being able to offer physical activity counselling to patients in their practice, being in the frontline and seeing patients in the earliest stages of chronic diseases. Research had shown that physicians’ own exercise habits influence their physical activity counselling of patients. Previous studies and systematic reviews had reported that healthcare providers who were physically active were more likely to counsel their patients on physical activity more compared to doctors who do not [7, 8]. These healthcare providers included physicians from various specialties and primary care providers (including general practice physicians, physician’s assistants, nurses and nurse practitioners) [8, 9]. Few studies looked into associations between physical activity levels of physicians and physical activity counselling to patients with specific chronic diseases [10, 11]. However, 2 studies were conducted amongst Japanese primary care physicians of different specialties and found similar associations between their physical activity levels with their physical activity counselling to patients with hyperlipidaemia, heart failure, hypertension and chronic kidney disease [12, 13].

In Malaysia, primary care management of patients with chronic diseases is offered both in government and private health clinics [14]. Private health clinics provide services in mainly urban areas [15]. Government health clinics are subsidized by the government whereas private clinics charge fees which are usually paid by patients, employers or insurance. Government clinics are manned by a team consisting of allied healthcare providers with doctors depending on the size of the clinics whereas private clinics are very often solo practices [16]. Private clinics rarely have supporting paramedical staff for assistance [17].

In contrast with other previous studies, a study conducted amongst PCDs in Malaysian government health clinics found no significant association between physical activity levels of PCDs and physical activity counselling to patients with chronic diseases. Only a third of PCDs offered physical activity counselling to 50% or more of their patients [18]. It is unknown whether physical activity levels of PCDs in private practice would influence their physical activity counselling practices as the working environment in Malaysian private practice differs from government clinics.

The general objective of this study is to determine if there is an association between physical activity levels of PCDs in private clinics with physical activity counselling to patients with major chronic diseases i.e. cardiovascular diseases, hypertension, type 2 diabetes mellitus, dyslipidaemia, obesity/metabolic syndrome, osteoarthritis/osteoporosis and bronchial asthma/chronic obstructive pulmonary disease. The specific objectives are a) to determine physical activity levels amongst primary care doctors in private practice from the Federal Territory of Kuala Lumpur, Selangor and Penang; b) to determine the frequency of initiation of exercise counselling by the participants to patients on chronic disease follow up; c) to determine the association between physical activity levels and confounding factors with frequency of initiation of exercise counselling to patients on chronic disease follow up. It is hoped that the findings from this study will help in formulating methods to improve physical counselling in patients with chronic diseases.

## Methods

### Study design and recruitment

This was a cross-sectional study conducted amongst PCDs in private practice from three states in Malaysia i.e. the Federal Territory of Kuala Lumpur (KL), Selangor and Penang from June to December 2016. These states were chosen as they were mainly urban with a high number of PCDs in private practice. All PCDs in private practice were included whereas those excluded were doctors who were away on prolonged leave, pregnant or who had conditions or disabilities limiting exercise during the data collection period.

PCDs from KL and Selangor were recruited from Academy of Family Physicians Malaysia (AFPM). Universal sampling was conducted as the authors were not able to access the AFPM’s member list in view of their confidentiality policy. Distribution of questionnaires was conducted by the AFPM’s administrative staff to the 728 PCDs registered members in KL and Selangor. Self-addressed, stamped envelopes were sent of which 192 were returned by post (10 were excluded as they did not fulfil the inclusion criteria). To further increase response rates, a reminder was given to participants of workshops attended by AFPM for their members in KL and Selangor and another 20 questionnaires were returned.

**Fig. 1:**
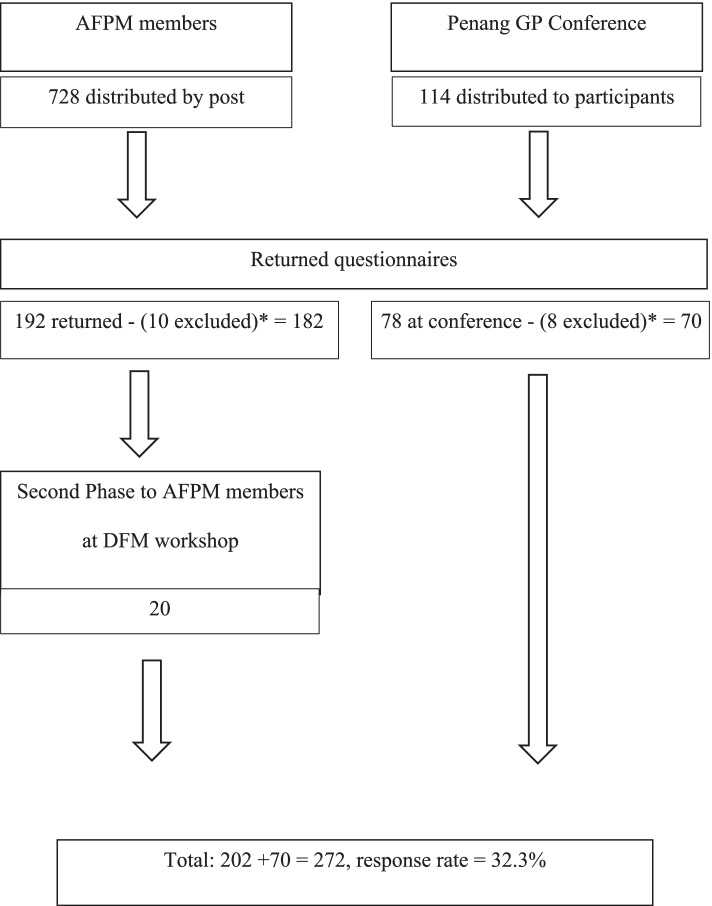
Summary of data collection process. (*): Participants were excluded as they did not fulfil the inclusion criteria

Private PCDs from Penang were recruited from those who attended the annual Penang General Practitioner’s Conference organized by the Malaysian Medical Association (MMA) in Penang. At the conference, 114 questionnaires were distributed to participants who fulfilled the inclusion criteria. A total of 78 questionnaires were returned in person or placed in designated labelled boxes at the end of each conference day. The recruitment process is summarized in Fig. 1. The sample size for this study was calculated from a total population size of PCDs in KL, Selangor and Penang of 842 (728 from Kuala Lumpur and Selangor and 114 from Penang respectively) using the Taro Yamane formula, [19] resulting in a recommended sample size of 271. Another an estimated 20% was added to account for non-responders and thefinal sample size required was 318.

### Questionnaire

The study tool consisted of a self-administered questionnaire (Appendix 1) divided into two sections. The first section was developed by the researchers and consisted of independent variables including sociodemographic data which were age, gender, height, weight, body mass index (BMI), smoking status, presence of chronic medical illnesses, years working, years in primary care, average number of patients seen per day and highest academic qualification (undergraduate/post-graduate).

The dependent variables measured comprised of frequency of exercise counselling to patients with chronic medical diseases (i.e., cardiovascular diseases, hypertension, type 2 diabetes mellitus, obesity/metabolic syndrome, dyslipidaemia, osteoarthritis/osteoporosis and bronchial asthma/COPD). These conditions were chosen as exercise has been shown to benefit these conditions [1]. The exercise counselling questions had a 5-point Likert scale and underwent prior content validation from a panel of 5 experts in primary care. Following their feedback, the questions were reconstructed until they were sufficiently clearly understood. Subsequently, face validation was done via a pilot study among 25 primary care doctors at the University Malaya Medical Centre and minor modifications were made to the final version.

The second section measured an independent variable which was physical activity levels of PCDs using the English version of the International Physical Activity Questionnaire (IPAQ) which has been validated in Malaysia [20]. The short version of the IPAQ which is more suitable for self-administration was used and consisted of 7 questions assessing time spent in activities of varying intensity which are walking, moderate-intensity and vigorous-intensity activities over the last 7 days. A score was calculated in metabolic equivalent of task (MET)-minutes per week which is a functional measurement of energy use. Based on the IPAQ scores, levels of physical activity were categorised into low, moderate and high levels of physical activity as per IPAQ guidelines [21].

### Statistical analyses

Data from this study was analysed using Statistical Package for Social Sciences (SPSS) software, Version 23.0. Descriptive statistics was for physical activity levels of private PCDs and frequency of initiation of exercise counselling to patients with chronic diseases. For exercise counselling frequency, Likert scores from 1–3 were classified as ‘rarely initiates’ and 4–5 as ‘often initiates’.

To determine associations between physical activity levels and other independent variables (confounding factors) with initiation of exercise counselling for each of the five chronic diseases, bivariate regression was conducted for categorical variables and continuous variables that were normally distributed.

For continuous variables that were not normally distributed, the Kruskal–Wallis test was done.

To identify significant independent variables, bivariate regression was conducted between demographic variables and initiation of exercise counselling for each chronic disease (‘Often initiates’ vs ‘Rarely initiates). For continuous variables that were not normally distributed, the Kruskal–Wallis test was performed. For each of the chronic diseases, independent variables with significant values (*p*-value ≤ 0.250) were included in subsequent multivariate analysis [22].

Multivariate analyses was used to determine the independent association between physical activity levels and exercise counselling. For this, all variables with a *p-* value of ≤ 0.25 were included in the analyses.

## Results

A total of 272 PCDs were included for analysis, with 202 from KL/Selangor and 70 from Penang. Response rates were 27.7% for KL/Selangor and 59.8% for Penang with an overall response rate of 32.3% (Fig. 1).

Table 1 shows the sociodemographic characteristics of respondents. The mean age of respondents was 34 ± 11 years, the majority (66.5%) of whom were female. The mean body mass index (BMI) of participants was 24.4 ± 0.26 kg/m^2^. The majority of participants (59.6%) were either overweight (36.4%) or obese (23.3%). Most of the participants (98.2%) were non-smokers.

**Table 1:** Sociodemographic characteristics of participants (*n* = 272

Variables	N (%)	Median (IQR)	Mean (SD)
Age			34 (11)
Gender			
Male	91 (33.5)		
Female	181 (66.5)		
BMI			
Underweigh	14 (5.1)		
Normal	96 (35.3)		24.4 ± 0.26
** Overweight**	**99 (36.4)**		
** Obese**	**63 (23.2**)		
Smoking			
Yes	5 (1.8)		
No	267 (98.2)		
Comorbidities			
Cardiovascular Diseases	3 (1.1)		
Hypertension	17 (6.3)		
Type 2 Diabetes Mellitus	13 (4.8)		
Obesity/Metabolic Syndrome	63 (23.2)		
Dyslipidaemia	39 (14.3)		
Bronchial Asthma/COPD	13 (4.8)		
Arthritis/Osteoporosis	19 (7.0)		
Years Working		8 (8.75)	
Years in Primary Care		5 (7.38)	
Highest Qualification			
Undergraduate	144 (52.9)		
Postgraduate	128 (47.1)		
Patients seen per day			40 (22.08)

The most common comorbidities in respondents were obesity/metabolic syndrome and dyslipidaemia at 23.2% and 14.3% respectively. The median total number of years working as a doctor was 8 (IQR 8.75) and median number of years working in primary care setting was 5 (IQR 7.38). Less than half (47.1%) had a post-graduate qualification. The mean number of patients seen on an average day was 40 ± 22.08.

Table 2 shows the frequency of initiation of exercise counselling to patients with chronic diseases. The majority of participants answered ‘always’ or ‘often’ for initiation of exercise counselling to patients with cardiovascular diseases (59.5%), hypertension (72.8%), type 2 diabetes mellitus (78.6%), obesity/metabolic syndrome (86.4%) and dyslipidaemia (81.6%).

**Table 2:** Frequency of initiation of exercise counselling to patients with chronic diseases (*n* = 272)

Types of chronic diseases	Frequency of initiation of exercise counselling, N (%)				
	**Never (0% of the time)**	**Rarely (25% of the time)**	**Sometimes (50% of the time)**	**Often (75% of the time)**	**Always (100% of the time)**
Cardiovascular Diseases	6(2.2)	41(15.1)	62(22.8)	104(38.2)	89(21.7)
Hypertension	2(0.7)	27(9.9)	45(16.5)	118(43.4)	80(29.4)
Type 2 Diabetes Mellitus	3(1.1)	18(6.6)	37(13.6)	110(40.4)	104(38.2)
Obesity/Metabolic Syndrome	0(0)	9(3.3)	28(3.3)	93(34.2)	142(52.2)
Dyslipidaemia	1(0.4)	13(4.8)	36(13.2)	124(45.6)	98(36)
Osteoarthritis&Osteoporosis	14 (5.1)	54 (19.9)	90 (33.1)	78 (28.7)	36 (13.2)
BA&COPD	29 (10.7)	73 (26.8)	90 (33.1)	54 (19.9)	26 (9.6)

Most participants (50%) had moderate levels of physical activity and 25.7% had low levels. Only 24.3% had high levels levels of physical activity.

Bivariate regression (Table 3) showed significant associations between physically active PCDs and increased initiation of exercise counselling to patients with cardiovascular diseases (OR = 3.200, *p* < 0.001, 95% CI 1.824 to 5.616) and hypertension (OR = 2.252, *p* = 0.046, 95% CI 1.016—4.989). There were no significant associations found between physically active PCDs and increased initiation of exercise counselling to patients with osteoarthritis/osteoporosis and BA/COPD.

**Table 3:** Simple logistic regression for association between initiation of exercise counselling and confounding factors

Initiation of exercise counselling for chronic disease (‘Often initiates’ vs ‘Rarely initiates’)	Confounding factors	Odds Ratio	95% CI	*p*-value
**Cardiovascular diseases**	Age	1.023	0.997 – 1.051	0.085
	Gender (Male)	0.643	0.368 – 1.093	0.101
	BMI	1.507	0.910 – 2.495	0.111
	Smoking (Yes vs No)	0.131	0.014 – 1.193	0.071
	Hypertension	2.679	0.750 – 9.566	0.129
	Years Working			0.098^b^
	Patients per day	0.982	0.971 – 0.994	0.002
**Hypertension**	Age	1.032	0.985 – 1.082	0.184
	Years Working			0.163^b^
	Years in Primary Care			0.195^b^
	Highest Qualification	3.117	1.284 – 7.567	0.012
	Patients per Day	0.104	0.972 – 1.003	0.987
**Type 2 diabetes mellitus**	Years Working			0.228^b^
	Highest Qualification	2.364	0.889 – 6.291	0.085
	Patients per day	0.977	0.960 – 0.994	0.007
**Obesity/metabolic syndrome**	Having hypertension	0.212	0.040 – 1.109	0.066
	Having dyslipidaemia	0.317	0.076 – 1.325	0.115
**Dyslipidaemia**	BMI	1.973	0.712 – 5.467	0.191
	Having dyslipidaemia	0.475	0.145 – 1.556	0.219

b = Kruskall-Wallis test

Multivariate regression (Table 4) revealed that for patients with cardiovascular diseases, PCDs who were physically active and non-smokers were more likely to initiate exercise (OR = 2.758, *p* = 0.001, 95% CI 1.518 – 5.012) and (OR = 11.488, *p* = 0.047 95% CI 1.031 – 127.986) respectively. PCDs with post-graduate qualifications were more likely to counsel hypertensive patients on exercise (OR = 2.806, *p* = 0.029, 95% CI 1.110 – 7.092). Initiation of exercise counselling to patients with cardiovascular diseases and diabetes mellitus was less likely with an increasing number of patients seen per day (OR = 0.987, *p* = 0.042, 95% CI 0.975 – 1.000) and (OR = 0.980, *p* = 0.027, 0.963 – 0.998) respectively.

**Table 4:** Multivariate regression between initiation of exercise counselling of chronic diseases with physical activity intensity and confounding factors

Initiation of exercise counselling for chronic diseases (‘Often initiates’ vs ‘Rarely initiates’)	Physical activity intensity and confounding factors	Odds Ratio	95% CI	*p*-value
**Cardiovascular diseases**	**Physically Active vs Physically Inactive**	**2.758**	**1.518 – 5.012**	**0.001**
	Age	0.980	0.861 – 1.116	0.761
	Gender (Male)	0.764	0.407 – 1.436	0.404
	BMI	1.350	0.776 – 2.349	0.288
	**Smoking (No vs Yes)**	**11.488**	**1.031 – 127.986**	**0.047**
	Hypertension	2.264	0.506 – 10.138	0.285
	Years Working	1.032	0.901 – 1.182	0.650
	**Patients per day**	**0.987**	**0.975 – 1.000**	**0.042**
**Hypertension**	Physically Active vs Physically Inactive	2.205	0.941 – 5.167	0.069
	Age	0.982	0.835 – 1.155	0.826
	Years Working	0.974	0.793 – 1.196	0.803
	Years in Primary Care	1.084	0.953 – 1.233	0.220
	**Highest Qualification**	**2.806**	**1.110 – 7.092**	**0.029**
	Patients per Day	0.993	0.976 – 1.010	0.411
**Type 2 diabetes mellitus**	Physically Active vs Physically Inactive	1.487	0.561—3.940	0.425
	Years Working	1.014	0.955 – 1.077	0.644
	Highest Qualification	2.057	0.734 – 5.768	0.170
	**Patients per day**	**0.980**	**0.963 – 0.998**	**0.027**
**Obesity/** **metabolic syndrome**	Physically Active vs Physically Inactive	2.556	0.646 – 10.110	0.181
	Having hypertension	0.267	0.042 – 1.708	0.163
	Having dyslipidaemia	0.461	0.094 – 2.265	0.341
**Dyslipidaemia**	BMI	2.036	0.731 – 5.673	0.174
	Having dyslipidaemia	0.454	0.137 – 1.499	0.195

## Discussion

The key findings of this study were that a majority of PCDs in private practice had moderate to high levels of physical activity and those who were physically active were more likely to initiate exercise counselling in patients with cardiovascular diseases.

### Physical activity levels of PCDs

In this study, 74.3% of PCDs in private practice were found to be physically active (moderate or high IPAQ scores). A larger proportion of our respondents appeared to be more physically than the general Malaysian adult population with the physically active at 66.5% (41.1% at a moderate intensity and 25.4% at high intensity) [2] and primary care physicians surveyed amongst Malaysian government clinics (51.4% were physically active) [18]. These findings were similar with other studies done in Northern Ireland (56.6% physically active) and Estonia (92%) [23, 24]. This is could be explained by doctors probably understanding the importance of being physically active. Conversely, doctors in Nigeria showed lower levels of physical activity (20.8%) than the general population (68.6—70.2%) [25].

### Frequency of exercise counselling

Most of the respondents in our study often initiated exercise counselling to patients with cardiovascular diseases, hypertension, type 2 diabetes mellitus, obesity/metabolic syndrome and dyslipidaemia. Similar trends were observed in other studies where in Northern Ireland, GPs reported comparable rates of exercise counselling to their patients with chronic diseases [26]. Morishita et al. found high frequencies of exercise counselling by PCDs to patients suffering from diabetes(90.3%) or dyslipidaemia(78.5%) [12]. With respect to hypertensive patients specifically, Hung et al. reported that 94.4% of primary care providers advised their patients to be physically active [10].

On the contrary, only about 32.8% of primary care doctors in government clinics in the Klang Valley provided exercise counselling to 50% or more of their patients with the majority to patients suffering from high cholesterol, obesity, type 2 diabetes mellitus and those who smoked or had a sedentary lifestyle [18]. The contrast here could be due to the difference in number of patients seen in government clinics with 58.8 ± 26.9 compared to 40 ± 22.08 patients per day in our study resulting in possibly insufficient time for exercise counselling. Other variables that may influence lower levels of physical activity could be attributed to differences in working environment.

### Variations in associations in physical activity levels of PCDs with physical activity counselling to patients with specific chronic diseases

Previous literature had shown an association between physical activity of doctors and exercise counselling where physically active doctors were more likely to provide exercise counselling [7, 27–29]. Our study’s findings do support that. However, we found that there was a significant association between physical activity intensity of PCDs in private practice and their exercise counselling but only to patients with cardiovascular diseases. There was a positive association for patients with other chronic diseases (hypertension, T2DM, obesity/metabolic syndrome, dyslipidaemia) though it was not significant. Other studies have shown significant associations between physical activity of PCDs and exercise counselling to patients with diabetes, hypertension and dyslipidaemia [12, 10]. The reasons our findings differ is unknown. Interestingly, no significant association was found between physical activity of primary care doctors in Malaysian government clinics and exercise counselling [18]. This could be influenced by other variables such as a higher number of patients seen in government facilities, resulting in less time for counselling. Aside from qualified doctors, medical students who practice active lifestyles too have a higher preponderance to advise patients on physical activity [28].

Apart from physical activity levels of PCDs, there are other factors that could influence physical activity counselling to patients with chronic diseases. In the multivariate analysis, participants in our study with post-graduate qualifications were also more likely to offer exercise counselling to hypertensive patients which could be due to the further training in exercise counselling that they may have received as post-graduates. A similar trend was seen in government clinics where family physicians with post-graduate degrees were more likely to offer exercise counselling compared doctors without post-graduate qualifications [18]. Similarly, trained physicians tend to spend more time offering physical activity counselling to patients than medical students or non-medically trained sports scientists [28]. Non-smokers were more likely to initiate exercise counselling in cardiovascular diseases and this was consistent with findings from Hung et al. that indicated non-smoking doctors were more likely to offer lifestyle intervention advise to hypertensive patients, including exercise [10]. This could possibly be in keeping with trends of healthcare personnel practicing a healthier lifestyle generally being more inclined to offer physical activity counselling to their patients [30–32].

### Strengths and limitations

This study to the best our knowledge currently is the first to look at associations between physical activity of general practitioners in the private sector from the federal territory of Kuala Lumpur, Selangor and Penang with initiation of exercise counselling to patients with specific chronic diseases. The findings from this study will pave the way for more research into the health and wellbeing of primary care doctors in private practice and their patient counselling practices.

A limitation of this study is that it used a self-reported questionnaire, therefore, it was subjected to some degree of reporting bias. Another limitation is the low response rate despite the researchers’ attempts in providing reminders.

Similar poor response rates had been seen in postal as well as online surveys done in other countries ranging from 31 to 68.4% [24, 26, 27, 33–40]. Some PCDs in private practice may not have the time to answer questionnaires when working in a solo practice.

Sampling primary care doctors who attended the GP conference in Penang was also subjected to bias as only doctors who were proactive in learning and continuous medical education (CME) sessions would have had the time as well as resources to spare to attend them. As this study was conducted amongst primary care doctors in private practice only in Kuala Lumpur, Selangor and Penang which are urban areas due to limitations in human resources and time, results from this study may not be generalizable to all primary care doctors practising in Malaysia. There may have been differences in physical activity counselling practices between PCDs from urban and rural locations which were otherwise uncaptured here. Our respondents were predominantly female too, possible skewing results as females tend more to counsel patients more than male physicians [34, 35] Furthermore, although the self-developed questions on initiation of exercise counselling to patients with chronic diseases underwent face and content validity; it would have been ideal for the questions to have undergone a full validation process.

### Recommendations

Increasing PCDs’ level of physical activity may increase their physical activity counselling to patients with cardiovascular diseases. Therefore, strategies to encourage doctors to stay active would be useful plans for the future such as involving and engaging PCDs in physical activity campaigns. In larger private practices, assigning a designated area for exercise or organizing fitness sessions for employees during protected time may be recommended.

Positive associations were seen in physical activity counselling given to patients with other types of chronic conditions such as hypertension, type 2 diabetes mellitus, dyslipidaemia and obesity/metabolic syndrome although it was not significant. There were no associations with counselling of patients with osteoarthritis and osteoporosis although these diseases would benefit from regular exercise. The reasons are unknown, hence, this warrants further research to explore these reasons.

## Conclusion

Most respondents in this study were physically active with 50% physically active at a moderate level and 24.3% at a high level.

Higher physical activity levels and being a non-smoker increased the initiation of exercise counselling to patients with cardiovascular diseases on follow-up visits. Private PCDs with postgraduate qualifications were more likely to initiate exercise counselling in hypertensive patients.

## Supplementary Information

**Additional file1:** **Appendix 1.** Fitness Questionnaire. Appendix 2. INTERNATIONAL PHYSICAL ACTIVITY QUESTIONNAIRE.

## Data Availability

The datasets generated and/or analysed during the current study are not publicly available due to limitations of ethical approval involving the patient data and anonymity but are available from the corresponding author on reasonable request.

## Abbreviations

PCD: Primary care doctor
PA: Physical activity
KL: Kuala Lumpur
BMI: Body mass index
