# Supplementary material for: Physically active primary care doctors are more likely to offer exercise counselling to patients with cardiovascular diseases: a cross-sectional study
Source: BMC Prim Care. 2022 Mar 29;23:59. doi: 10.1186/s12875-022-01657-3 (PMC8966347; doi:10.1186/s12875-022-01657-3)
Supplement: Supplementary file 1 — Additional file1: Appendix 1. Fitness Questionnaire. Appendix 2. INTERNATIONAL PHYSICAL ACTIVITY QUESTIONNAIRE. [file 12875_2022_1657_MOESM1_ESM.docx]

## Appendix 1

**Fitness Questionnaire**

***Please √ / answer the following questions.***

1. How old are you? _____ years
2. Gender (please tick where applicable) Male ( ) Female ( )
3. Height: _____cms
4. Weight: _____ kgs
5. Body Mass Index (BMI): _____
6. Do you smoke? (Please tick where applicable) Yes ( ) No ( )

(If you answered ‘Yes’, please specify the number of cigarettes per day _____)

1. Do you have any of the following chronic medical conditions? (Please tick where applicable)
   1. Cardiovascular Diseases (Heart Disease and Stroke) Yes ( ) No ( )
   2. Hypertension Yes ( ) No ( )
   3. Type 2 Diabetes Mellitus Yes ( ) No ( )
   4. Obesity/Metabolic Syndrome Yes ( ) No ( )
   5. Dyslipidaemia Yes ( ) No ( )
   6. Arthritis/Osteoporosis Yes ( ) No ( )
   7. Bronchial Asthma/COPD Yes ( ) No ( )

1. How many years have you been working since graduating? _____
2. What is your highest qualification? (Please √ where applicable)
   1. Undergraduate degree (i.e. MBBS, MD, MBChB) _____
   2. Postgraduate degree (i.e. MFamMed, FRACGP, DFM) ____
3. How many patients do you see on average per day? _____

***For the following questions 11 and 12, please choose an answer from a scale of 1 to 5 and circle where applicable.***

**11a) For the following chronic disease follow-up visits, how frequently do you counsel your patients on exercise IN A WEEK?**

1. **Cardiovascular Diseases (Heart Disease and Stroke)**

| Never  (0% of the time) | Rarely  (25% of the time) | Sometimes  (50%of the time) | Often  (75%of the time) | Always  (100% of the time) |
| --- | --- | --- | --- | --- |
| **1** | **2** | **3** | **4** | **5** |

1. **Hypertension**

| Never  (0% of the time) | Rarely  (25% of the time) | Sometimes  (50%of the time) | Often  (75%of the time) | Always  (100% of the time) |
| --- | --- | --- | --- | --- |
| **1** | **2** | **3** | **4** | **5** |

1. **Type 2 Diabetes Mellitus**

| Never  (0% of the time) | Rarely  (25% of the time) | Sometimes  (50%of the time) | Often  (75%of the time) | Always  (100% of the time) |
| --- | --- | --- | --- | --- |
| **1** | **2** | **3** | **4** | **5** |

1. **Obesity / Metabolic Syndrome**

| Never  (0% of the time) | Rarely  (25% of the time) | Sometimes  (50%of the time) | Often  (75%of the time) | Always  (100% of the time) |
| --- | --- | --- | --- | --- |
| **1** | **2** | **3** | **4** | **5** |

1. **Dyslipidaemia**

| Never  (0% of the time) | Rarely  (25% of the time) | Sometimes  (50%of the time) | Often  (75%of the time) | Always  (100% of the time) |
| --- | --- | --- | --- | --- |
| **1** | **2** | **3** | **4** | **5** |

1. **Arthritis (Including Osteoarthritis, Chronic Back Pain) and Osteoporosis**

| Never  (0% of the time) | Rarely  (25% of the time) | Sometimes  (50%of the time) | Often  (75%of the time) | Always  (100% of the time) |
| --- | --- | --- | --- | --- |
| **1** | **2** | **3** | **4** | **5** |

1. **Bronchial Asthma/Chronic Obstructive Pulmonary Disease**

| Never  (0% of the time) | Rarely  (25% of the time) | Sometimes  (50%of the time) | Often  (75%of the time) | Always  (100% of the time) |
| --- | --- | --- | --- | --- |
| **1** | **2** | **3** | **4** | **5** |

**11b) What percentage of your patients on the following chronic disease follow up visits do you initiate counselling on exercise (before a patient asks about it) IN A WEEK?**

1. **Cardiovascular Diseases (Heart Disease and Stroke)**

| None  (0% of my patients) | Some  (25% of my patients) | Half  (50% of my patients) | Most  (75% of my patients) | All  (100% of my patients) |
| --- | --- | --- | --- | --- |
| **1** | **2** | **3** | **4** | **5** |

1. **Hypertension**

| None  (0% of my patients) | Some  (25% of my patients) | Half  (50% of my patients) | Most  (75% of my patients) | All  (100% of my patients) |
| --- | --- | --- | --- | --- |
| **1** | **2** | **3** | **4** | **5** |

1. **Type 2 Diabetes Mellitus**

| None  (0% of my patients) | Some  (25% of my patients) | Half  (50% of my patients) | Most  (75% of my patients) | All  (100% of my patients) |
| --- | --- | --- | --- | --- |
| **1** | **2** | **3** | **4** | **5** |

1. **Obesity / Metabolic Syndrome**

| None  (0% of my patients) | Some  (25% of my patients) | Half  (50% of my patients) | Most  (75% of my patients) | All  (100% of my patients) |
| --- | --- | --- | --- | --- |
| **1** | **2** | **3** | **4** | **5** |

1. **Dyslipidaemia**

| None  (0% of my patients) | Some  (25% of my patients) | Half  (50% of my patients) | Most  (75% of my patients) | All  (100% of my patients) |
| --- | --- | --- | --- | --- |
| **1** | **2** | **3** | **4** | **5** |

1. **Arthritis (Osteoarthritis and Chronic Back Pain) and Osteoporosis**

| None  (0% of my patients) | Some  (25% of my patients) | Half  (50% of my patients) | Most  (75% of my patients) | All  (100% of my patients) |
| --- | --- | --- | --- | --- |
| **1** | **2** | **3** | **4** | **5** |

1. **Bronchial Asthma/ Chronic Obstructive Pulmonary Disease**

| None  (0% of my patients) | Some  (25% of my patients) | Half  (50% of my patients) | Most  (75% of my patients) | All  (100% of my patients) |
| --- | --- | --- | --- | --- |
| **1** | **2** | **3** | **4** | **5** |

**12. How prepared are you to initiate exercise counselling in patients on chronic disease follow-up visits (E.g. Patients with Cardiovascular Diseases, Hypertension, Obesity/Metabolic Syndrome, Type 2 Diabetes Mellitus, Dyslipidaemia, Arthritis/Osteoporosis, Bronchial Asthma/Chronic Obstructive Pulmonary Disease)?**

|  |  | Strongly  Disagree | Disagree | Uncertain | Agree | Strongly Agree |
| --- | --- | --- | --- | --- | --- | --- |
| **12(a)** | **I am confident in my abilities to counsel my patients on exercise.** | **1** | **2** | **3** | **4** | **5** |
| **12(b)** | **I am comfortable talking about exercise with my patients.** | **1** | **2** | **3** | **4** | **5** |
| **12(c)** | **I have adequate knowledge on exercise to counsel my patients about it.** | **1** | **2** | **3** | **4** | **5** |

## Appendix 2

# INTERNATIONAL PHYSICAL ACTIVITY QUESTIONNAIRE

We are interested in finding out about the kinds of physical activities that people do as part of their everyday lives. The questions will ask you about the time you spent being physically active in the **last 7 days**. Please answer each question even if you do not consider yourself to be an active person. Please think about the activities you do at work, as part of your house and yard work, to get from place to place, and in your spare time for recreation, exercise or sport.

Think about all the **vigorous** activities that you did in the **last 7 days**. **Vigorous** physical activities refer to activities that take hard physical effort and make you breathe much harder than normal. Think *only* about those physical activities that you did for at least 10 minutes at a time.

1. During the **last 7 days**, on how many days did you do **vigorous**

physical activities like heavy lifting, digging, aerobics, or fast bicycling?

**days per week**

No vigorous physical activities  ***Skip to question 3***

1. How much time did you usually spend doing **vigorous** physical activities on one of those days?

**hours per day**

**minutes per day**

Don’t know/Not sure

SHORT LAST 7 DAYS SELF-ADMINISTERED version of the IPAQ. Revised August 2002.

Think about all the **moderate** activities that you did in the **last 7 days**. **Moderate** activities refer to activities that take moderate physical effort and make you breathe somewhat harder than normal. Think only about those physical activities that you did for at least 10 minutes at a time.

1. During the **last 7 days**, on how many days did you do **moderate** physical activities like carrying light loads, bicycling at a regular pace, or doubles tennis? Do not include walking.

**days per week**

No moderate physical activities  ***Skip to question 5***

1. How much time did you usually spend doing **moderate** physical activities on one of those days?

**hours per day**

**minutes per day**

Don’t know/Not sure

SHORT LAST 7 DAYS SELF-ADMINISTERED version of the IPAQ. Revised August 2002.

Think about the time you spent **walking** in the **last 7 days**. This includes at work and at home, walking to travel from place to place, and any other walking that you have done solely for recreation, sport, exercise, or leisure.

1. During the **last 7 days**, on how many days did you **walk** for at least 10 minutes at a time?

**days per week**

No walking  ***Skip to question 7***

1. How much time did you usually spend **walking** on one of those days?

**hours per day**

**minutes per day**

Don’t know/Not sure

SHORT LAST 7 DAYS SELF-ADMINISTERED version of the IPAQ. Revised August 2002.

The last question is about the time you spent **sitting** on weekdays during the **last 7 days**. Include time spent at work, at home, while doing course work and during leisure time. This may include time spent sitting at a desk, visiting friends, reading, or sitting or lying down to watch television.

1. During the **last 7 days**, how much time did you spend **sitting** on a **week day**?

_ **hours per day**

_ **minutes per day**

Don’t know/Not sure

**This is the end of the questionnaire, thank you for participating.**

SHORT LAST 7 DAYS SELF-ADMINISTERED version of the IPAQ. Revised August 2002.
